# Reference Genes for Accurate Transcript Normalization in Citrus Genotypes under Different Experimental Conditions

**DOI:** 10.1371/journal.pone.0031263

**Published:** 2012-02-09

**Authors:** Valéria Mafra, Karen S. Kubo, Marcio Alves-Ferreira, Marcelo Ribeiro-Alves, Rodrigo M. Stuart, Leonardo P. Boava, Carolina M. Rodrigues, Marcos A. Machado

**Affiliations:** 1 Laboratório de Biotecnologia, Centro de Citricultura Sylvio Moreira, Cordeirópolis-São Paulo, Brazil; 2 Instituto de Biologia, Universidade Estadual de Campinas, Campinas-São Paulo, Brazil; 3 Laboratório de Genética Molecular Vegetal, Departamento de Genética, Instituto de Biologia, Universidade Federal do Rio de Janeiro, Rio de Janeiro, Brazil; 4 Instituto Oswaldo Cruz, Fundação Oswaldo Cruz, Rio de Janeiro, Brazil; East Carolina University, United States of America

## Abstract

Real-time reverse transcription PCR (RT-qPCR) has emerged as an accurate and widely used technique for expression profiling of selected genes. However, obtaining reliable measurements depends on the selection of appropriate reference genes for gene expression normalization. The aim of this work was to assess the expression stability of 15 candidate genes to determine which set of reference genes is best suited for transcript normalization in citrus in different tissues and organs and leaves challenged with five pathogens (*Alternaria alternata*, *Phytophthora parasitica*, *Xylella fastidiosa* and *Candidatus* Liberibacter asiaticus). We tested traditional genes used for transcript normalization in citrus and orthologs of *Arabidopsis thaliana* genes described as superior reference genes based on transcriptome data. geNorm and NormFinder algorithms were used to find the best reference genes to normalize all samples and conditions tested. Additionally, each biotic stress was individually analyzed by geNorm. In general, *FBOX* (encoding a member of the F-box family) and *GAPC2* (GAPDH) was the most stable candidate gene set assessed under the different conditions and subsets tested, while *CYP* (cyclophilin), *TUB* (tubulin) and *CtP* (cathepsin) were the least stably expressed genes found. Validation of the best suitable reference genes for normalizing the expression level of the *WRKY70* transcription factor in leaves infected with *Candidatus* Liberibacter asiaticus showed that arbitrary use of reference genes without previous testing could lead to misinterpretation of data. Our results revealed *FBOX*, *SAND* (a SAND family protein), *GAPC2* and *UPL7* (ubiquitin protein ligase 7) to be superior reference genes, and we recommend their use in studies of gene expression in citrus species and relatives. This work constitutes the first systematic analysis for the selection of superior reference genes for transcript normalization in different citrus organs and under biotic stress.

## Introduction

Real-time reverse transcription PCR (RT-qPCR) has emerged as the most widely used method to quantify changes in gene expression profiles in response to developmental transitions and environmental changes in plants. In comparison to classical methods used to measure transcript abundance, the main advantages of RT-qPCR are its higher sensitivity and specificity, even when limited amounts of RNA are available [1]. Nevertheless, to ensure reproducible and accurate quantitative expression measures, it is necessary to normalize the expression levels of target genes using suitable reference genes. An ideal reference gene should be stably expressed among samples, including those from different tissues and cell types, developmental stages, and treatment conditions [2]–[5]. Because there is no gene that meets all requirements for every experimental condition, a systematic validation of the stability of expression of candidate reference genes should be conducted in preliminary experiments assessing their usefulness for gene expression normalization [2], [6]. Gene expression analysis in citrus in different tissues and organs and under several experimental conditions has relied on the use of traditional housekeeping genes, such as *ACTIN* [7]–[12]; *EF1-α* [13]–[16]; *TUBULIN* [17] *GAPDH* [18], and *18S rRNA* [19] as reference genes, but with no previous testing of the stability of expression. It is generally assumed that housekeeping genes encoding proteins required for basal cell activities, such as central carbon metabolism, protein translation, cytoskeleton maintenance, and protein turnover, are expressed uniformly in different tissues and organs [6]. However, under many conditions, the level of transcript expressed from such genes was not stable, which may have led to the misinterpretation of results [20]–[24]. Statistical algorithms such as geNorm [25] and NormFinder [26] have been recently used to identify the best reference genes for RT-qPCR data normalization in a given set of biological samples. These algorithms have been used for assessing the expression stability of candidate reference genes across a variety of tissues and organs, developmental stages, biotic and abiotic stresses and cultivars in many plant species such as grapevine [27]; rice [28], [29]; tomato [30]; soybean [31]; coffee [32]; brachiaria grass [33]; cotton [34]; eucalyptus [35]; cucumber [36] and petunia [37]. To date, only three studies relying on RT-qPCR analysis in citrus have validated candidate reference genes for transcript normalization. These studies were limited to a few test conditions such as drought [38], leaf tissues of different citrus genotypes and a few organs [39], and *Phytophthora parasitica* infection [40]. Citrus is one of the most important commercial and nutritional fruit crops in the world. From a scientific standpoint, citrus has proven a valuable resource for studying distinctive aspects of development and physiology such as non-climacteric fruit development, apomixis, gametophytic self- and cross-incompatibility, juvenility, deciduousness versus evergreen foliage, dormancy, seasonality, and root-shoot interaction [41]. In addition, draft genomic sequences of the sweet orange (*Citrus sinensis* L. Osb.) and clementine mandarin (*C. clementina*) are now available (http://www.phytozome.net/clementine.php). The two reference genomes will greatly facilitate studies of functional genomics for genetic improvement in citrus and provide the opportunity to explore peculiar characteristics that cannot be easily addressed in herbaceous model plants such as *Arabidopsis thaliana* [42], [43]. Therefore, the identification of reliable reference genes in citrus will be crucial to allow accurate measurements for gene expression analysis in functional genomics studies. In this study, we aimed to identify potential reference genes suitable for transcript normalization in different samples, tissues, and organs of citrus under different treatments and then validate them. These reference genes will enable more accurate and reliable RT-qPCR normalization for gene expression studies in citrus.

## Results

### Identification of candidate citrus reference genes

**Figure 1:**
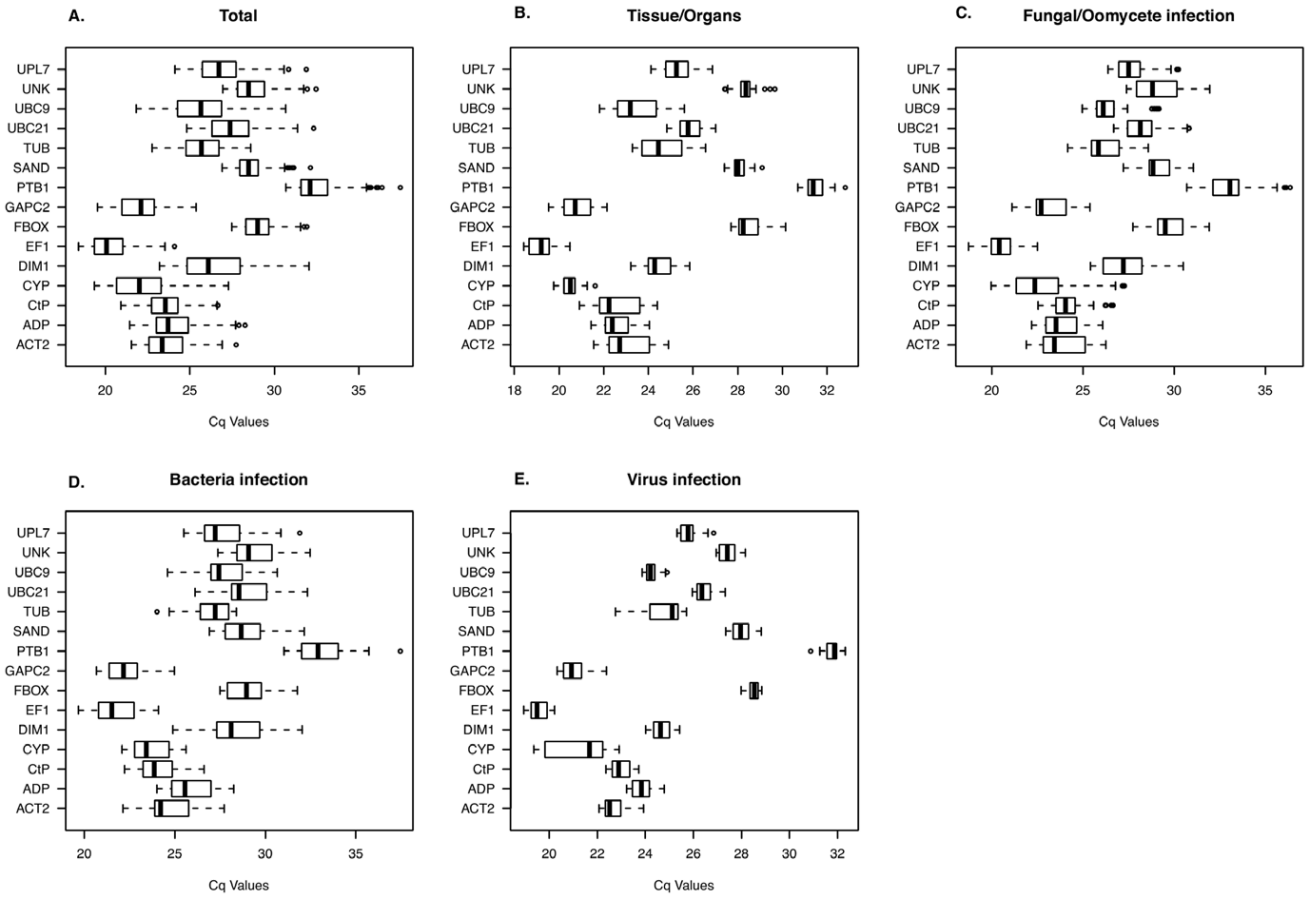
Expression levels of candidate reference genes in different experimental sets. Box plot graphs of Cq values for each reference gene tested in all citrus samples and subsets. Cq values are inversely proportional to the amount of template and are shown as the first and third quartile. Vertical lines indicate range of values, and median values are indicated by the black lines. Circles indicate outliers. (a) Total citrus samples examined, (b) Different citrus tissues or organs, (c) Fungal or oomycete stress, (d) Bacterial stress and (e) Viral stress.

In order to identify suitable citrus reference genes, 15 candidates were chosen from three sources: traditional housekeeping genes frequently used for transcript normalization in citrus; citrus homologues to superior reference genes selected from *Arabidopsis* transcriptome microarray data [21], and reference genes tested in Swingle citrumelo under drought stress [38]. Gene names, accession numbers, descriptions and functions according to The Arabidopsis Initiative Resource (TAIR) are listed in Table 1. To select citrus coding sequences, a BLASTN search using *Arabidopsis* reference genes as queries was performed in the CitEST and Harvest Citrus databases. All putative citrus homolog sequences showed very high similarities (see Table 1). Primers amplified a single PCR product as confirmed on a 2% agarose gel (Figure S1). The stability of expression of the candidate genes was assessed by RT-qPCR in a set of 38 samples grouped into six experiments. The first experimental set was composed of different organs and flower developmental stages from healthy plants, and the remainder were composed of five biotic stresses, including some discrete infection times: two bacterial species (*Xylella* and *Candidatus* Liberibacter asiaticus); one fungus (*Alternaria alternata*); one oomycete (*Phytophthora parasitica*); and one virus (*Citrus leprosis virus C*) (Figure 1). In addition, five species of *Citrus* (*C. sinensis* L. Osb., *C. reticulata* Blanco, *C. clementina*, *C. reshni* hort. ex Tanaka and *C. sunki* (Hayata) hort. ex Tanaka) a related specie (*Poncirus trifoliata*) and a hybrid (Murcott tangor (*C. sinensis×C. reticulata*)), were included in the set of biological samples evaluated.

**Table 1:** Citrus candidate reference gene description and comparison with Arabidopsis orthologs.

AGI^a^	Citrus Unigene^b^	Gene symbol	Gene name	tBLASTN (E-value)	Identity (%)
At2g28390	CAS-CS-112545	*SAND*	SAND family protein	1e-176	78%
	CAS-PT-305712			7e-97	77%
At5g08290	CAS-CS-106114	*DIM1*	DIM1 homolog/YLS8	1e-119	84%
	CAS-CR-206690			1e-118	83%
	CAS-PT-303795			1e-116	86%
At2g32170	CAS-CS-102441	N/A	Unknown protein	1e-109	81%
	CAS-PT-306913			1e- 94	80%
At5g15710	CAS-PT-306416	*FBOX*	F-box family protein	1e-131	79%
At3g53090	CAS-CS-110985	*UPL7*	Ubiquitin-protein ligase 7	0	78%
At5g25760	CAS-CS-101970	*UBC21*	Ubiquitin-conjugating enzyme 21	8e-93	79%
	CAS-CR-202884			1e-94	79%
At3g01150	CAS-CS-108488	*PTB1*	Polypyrimidine tract-binding protein 1	1e-150	80%
At1g13440	CAS-CS-106805	*GAPC2*	Glyceraldehyde-3-phosphate dehydrogenase C2	0	85%
	CAS-CR-204567			0	84%
	CAS-PT-300594			0	84%
At4g27960	CAS-CS-103344	*UBC9*	Ubiquitin conjugating enzyme 9	1e-123	84%
	CAS-CR-208944			1e -128	84%
	CAS-PT-301931			1e -107	81%
At3g18780	CAS-CS-103225	*ACT2*	Actin-2	0	83%
	CAS-CR-200290			0	83%
	CAS-PT-300172			0	83%
At5g60390	CAS-CS-107366	*EF-1α*	Elongation factor 1-alpha	0	86%
	CAS-CR-206424			0	87%
	CAS-PT-304425			0	87%
At1G20010	CAS-CS-106408	*TUB*	beta-Tubulin	0	83%

Eleven of the fifteen candidate citrus reference genes were selected according to their similarity to reference genes identified in *Arabidopsis*. Citrus sequences were retrieved from the citrus database (CitEST). Sequences used to design primer pairs for *ADP-ribosylation factor* (*ADP*), *cathepsin* (*CtP*) and *cyclophilin* (*CYP*) were retrieved from HarvEST Citrus according to Carvalho et al. (2010).

a *Arabidopsis* Gene Initiative (AGI) locus identifier number.

b Unigene identifier according to CitEST database. Abbreviations: CS- *Citrus sinensis*; CR- *C. reticulata*; PT- *Poncirus trifoliata*.

### RT-qPCR analysis

RT-qPCR was optimized for each primer pair, and two or three independent biological samples under each experimental condition were evaluated in technical triplicates (see Table S1). Melting curve analysis confirmed the presence of a single PCR product from all samples with no primer-dimers (Figure S2). Amplification efficiency was estimated using the *Miner* tool; the values ranged from 92 to 98%, except for *GAPC2* (84.5%) and *PTB1* (79.3%) (Table S1). Cycle quantification for each reaction, determined by the maximum point of the second derivative curve, was also estimated using *Miner*. Mean Cq values and their standard deviation are presented in Figure 1 for each transcript amplified from each biological replicate. Average Cq values ranged from 20.3 to 32.5; *Ef1-α* presented the highest and *PTB1* transcripts the lowest expression level among all samples (Figure 1a).

### Expression stability analysis

**Figure 2:**
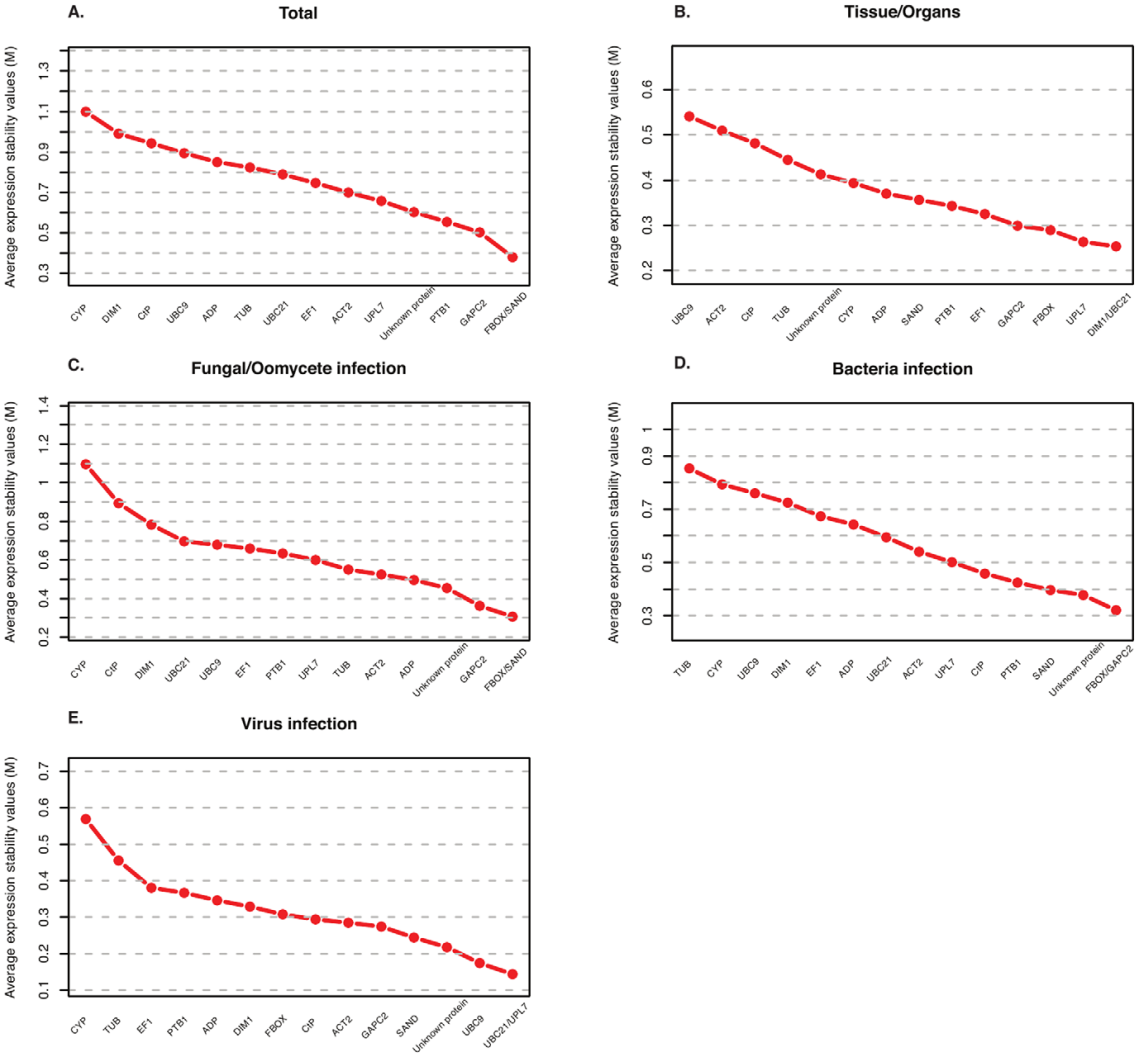
Average expression stability values (M) calculated by geNorm. M values of the remaining candidate citrus reference genes during stepwise exclusion of the least stable citrus reference gene in the different subsets. The ranking of the reference genes is in Table 2. A lower M value indicates more stable expression. (a) Total citrus samples examined, (b) Different citrus tissues or organs, (c) Fungal or oomycete stress, (d) Bacterial stress and (e) Viral stress.

**Figure 3:**
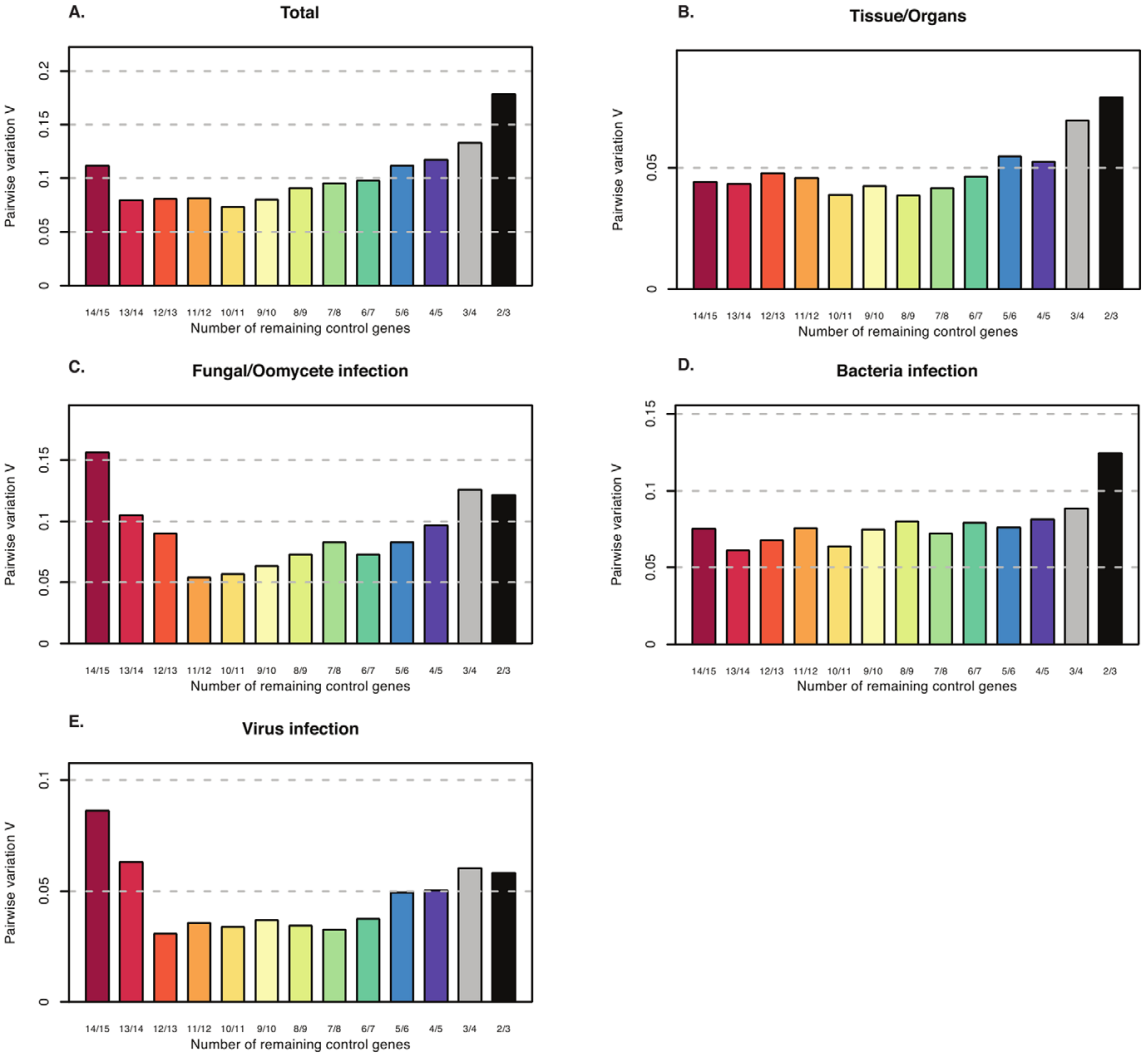
Pairwise variation (V) to determine the optimal number of reference genes for each subset. (a) Total citrus samples examined, (b) Different citrus tissues or organs, (c) Fungal or oomycete stress, (d) Bacterial stress and (e) Viral stress. The ranking of the reference genes is in Table 2.

In order to find the most stably expressed genes suitable for citrus RT-qPCR normalization, we assessed the stability of expression of 15 candidate genes using the pairwise variation in expression stability implemented in geNorm v3.5 [25]. geNorm estimates two parameters to find the best-suited reference genes: the average expression stability value (*M* value), and the pairwise variation (*V_n/n+1_*). The *M* value is estimated by the pairwise difference between a particular reference gene and all others. At the first step, the *M* value for all candidate genes is calculated. At the second, the reference gene with the lowest stability of expression (highest *M* value) is excluded and a new *M* value is calculated with the remaining reference genes. Moreover, the pairwise variation (*V_n/n+1_*) will determine the need for inclusion of additional reference genes in the normalization factor to produce accurate and reliable normalization. Quantities (Q) of the 15 candidate reference genes calculated for each biological sample were used in geNorm to calculate *M* stability values. At each step, reference genes with the lowest stability of transcript accumulation (the highest *M*) were excluded until the two most stably expressed genes remained. Figure 2a and Table 2 display the *M* values of reference genes examined when all samples were considered. We found that the *FBOX* and *SAND* genes were considered the most stably expressed overall (*M* = 0.39), while *CYP* was the least (*M* = 1.1). In addition, all 15 genes showed acceptable expression stabilities (*M*≤1), as observed by Hellemans and coworkers in heterogeneous samples [43]. We also calculated the optimal number of reference genes needed for a more reliable normalization in geNorm (V_n/n+1_). Taking into account the entire dataset and considering a cut-off (V_n/n+1_≤0.15, *FBOX*, *SAND* and *GAPC2* (V_3/4_ = 0.13) would be necessary for proper normalization (Figure 3a). In contrast, *UPL7* was determined by NormFinder to be the most stable reference gene, whereas *CYP* was again ranked as the most variable. *SAND*, *FBOX* and *GAPC2* were ranked in positions 6, 7 and 4, respectively, according to NormFinder (Table 3). Evaluating the six least stable reference genes in both geNorm and NormFinder, we found that *TUB*, *ADP*, *UBC9*, *Ctp*, *DIM1* and *CYP* were ranked in the same positions. Although the results obtained by the two algorithms seem to be divergent in selecting reference genes suitable for normalizing all citrus sample sets, our results reveal that at least five more stable reference genes (*FBOX*, *SAND*, *UPL7*, *PTB1* or *GAPC2*) could be selected. Besides the analysis in geNorm with the total sample sets, we divided the entire dataset into four subsets that were reanalyzed, and also analyzed by the model-based approach for estimation of expression variation proposed by NormFinder, which estimates the stability of gene expression based on the comparison between inter- and intra-group variability [26]. Analysis of the best reference genes in each experimental subset showed some differences (Table 2). Eight different tissues or organs composed of vegetative (leaf, branch), reproductive (flower bud at two stages of development, flower and fruitlet), and meristem samples were grouped in subset 1. The *DIM1*/*UBC21* pair was ranked as the most stable reference pair of genes by geNorm and *GAPC2* as the most stable gene by NormFinder (Figure 2b and Tables 2, 3). The best combination of two genes according to NormFinder was *GAPC2* and *UPL7*.

**Table 2:** Citrus reference genes ranked according to their expression stability as determined by geNorm.

Ranking	Total		Tissue/organ		Fungal/oomycete stress		Bacterial stress		Viral stress	
	Gene	Stability value (M)	Gene	Stability value (M)	Gene	Stability value (M)	Gene	Stability value (M)	Gene	Stability value (M)
1	*FBOX*	0.379	*DIM1*	0.254	*FBOX*	0.307	*FBOX*	0.321	*UBC21*	0.144
1	*SAND*	0.379	*UBC21*	0.254	*SAND*	0.307	*GAPC2*	0.321	*UPL7*	0.144
2	*GAPC2*	0.502	*UPL7*	0.264	*GAPC2*	0.362	*UNK	0.378	*UBC9*	0.174
3	*PTB1*	0.554	*FBOX*	0.290	*UNK	0.454	*SAND*	0.397	*UNK	0.218
4	*UNK	0.602	*GAPC2*	0.299	*ADP*	0.496	*PTB1*	0.425	*SAND*	0.244
5	*UPL7*	0.657	*EF1*	0.325	*ACT2*	0.525	*CtP*	0.459	*GAPC2*	0.274
6	*ACT2*	0.699	*PTB1*	0.343	*TUB*	0.550	*UPL7*	0.501	*ACT2*	0.285
7	*EF1*	0.746	*SAND*	0.356	*UPL7*	0.600	*ACT2*	0.541	*CtP*	0.294
8	*UBC21*	0.789	*ADP*	0.370	*PTB1*	0.634	*UBC21*	0.595	*FBOX*	0.308
9	*TUB*	0.823	*CYP*	0.394	*EF1*	0.659	*ADP*	0.643	*DIM1*	0.329
10	*ADP*	0.850	*UNK	0.413	*UBC9*	0.679	*EF1*	0.674	*ADP*	0.346
11	*UBC9*	0.894	*TUB*	0.445	*UBC21*	0.696	*DIM1*	0.724	*PTB1*	0.367
12	*CtP*	0.943	*CtP*	0.481	*DIM1*	0.783	*UBC9*	0.760	*EF1*	0.381
13	*DIM1*	0.990	*ACT2*	0.509	*CtP*	0.894	*CYP*	0.793	*TUB*	0.455
14	*CYP*	1.099	*UBC9*	0.541	*CYP*	1.096	*TUB*	0.853	*CYP*	0.569

*UNK: Unknown protein.

M stability values calculated by geNorm considering all tissues and experimental conditions (total) and each subset (tissue or organ; fungal or oomycete stress; bacterial stress and viral stress). M values are ranked from the most stable pair of genes to the least stable gene.

**Table 3:** Candidate genes ranked according to their expression stability as determined by NormFinder.

Ranking	Total		Tissue/organ		Fungal/oomycete stress		Bacterial stress		Viral stress	
	Gene	Stability value	Gene	Stability value	Gene	Stability value	Gene	Stability value	Gene	Stability value
1	*UPL7*	0.094	*GAPC2*	0.006	*FBOX*	0.040	*ACT2*	0.041	*UBC9*	0.010
2	*EF1*	0.105	*FBOX*	0.015	*GAPC2*	0.066	*PTB1*	0.069	*DIM1*	0.010
3	*PTB1*	0.115	*ADP*	0.027	*SAND*	0.069	*ADP*	0.090	*FBOX*	0.011
4	*GAPC2*	0.179	*DIM1*	0.028	*UPL7*	0.070	*UBC21*	0.092	*ADP*	0.016
5	*UBC21*	0.183	*UBC21*	0.030	*EF1*	0.080	*EF1*	0.102	*PTB1*	0.023
6	*SAND*	0.187	*UPL7*	0.034	*ADP*	0.136	*UNK	0.154	*UPL7*	0.028
7	*FBOX*	0.215	*EF1*	0.050	*PTB1*	0.142	*SAND*	0.162	*UBC21*	0.031
8	*ACT2*	0.233	*PTB1*	0.059	*TUB*	0.165	*UPL7*	0.167	*EF1*	0.034
9	*UNK	0.251	*SAND*	0.074	*UBC21*	0.197	*CtP*	0.175	*UNK	0.044
10	*TUB*	0.253	*TUB*	0.103	*UBC9*	0.202	*GAPC2*	0.188	*SAND*	0.054
11	*ADP*	0.291	*UNK	0.116	*UNK	0.206	*FBOX*	0.273	*CtP*	0.087
12	*UBC9*	0.404	*CYP*	0.119	*ACT2*	0.292	*CYP*	0.281	*ACT2*	0.107
13	*CtP*	0.458	*CtP*	0.146	*DIM1*	0.655	*UBC9*	0.305	*GAPC2*	0.120
14	*DIM1*	0.564	*ACT2*	0.160	*CtP*	0.820	*DIM1*	0.310	*TUB*	0.298
15	*CYP*	1.326	*UBC9*	0.206	*CYP*	2.614	*TUB*	0.597	*CYP*	0.796
Best pair	*UPL7/PTB1*		*GAPC2/UPL7*		*FBOX/UPL7*		*ACT2/PTB1*		*DIM1/FBOX*	
Stability value	0.110		0.094		0.166		0.214		0.077	

*UNK: Unknown protein.

Stability values are listed from the most stable to the least stable gene.

Despite the differences, when comparing the *M* value calculated for the *DIM1* and *UBC21* genes in geNorm, the exclusion of the *GAPC2* or *UPL7* genes displayed low variation (0.05 and 0.001, respectively) in the average expression stability value. Both pairs of reference genes (*DIM1/UBC21* and *GAPC2/UPL7*) can be used to normalize the expression of target genes in different tissues or organs of citrus. Analysis of the pairwise variation revealed that the *DIM1* and *UBC21* genes (V_2/3_ = 0.079) would be sufficient for normalizing gene expression (Figure 3b). The *CtP*, *ACT2* and *UBC9* genes were considered the most variable reference genes using both algorithms.

The second subset assessed was composed of leaves infected with *A. alternata* (6 and 12 h post-inoculation), and leaves collected 48 h post-inoculation with *P. parasitica*. The *FBOX*/*SAND* pair was selected as the least variable among all reference genes by geNorm (Figure 2c and Table 2). In NormFinder, *FBOX* was the most stable, followed by *GAPC2* and *SAND* (see Table 3). *DIM1*, which was selected as the most stable in subset 1, was one of the three least stably expressed in subset 2. Furthermore, when the two experimental conditions (fungus and oomycete) were analyzed by geNorm separately, and considering a cutoff of *M*≤0.5, any reference gene except *UBC9* (*M* = 0.55), and at least eight reference genes could be selected as good candidate reference genes for transcript normalization in citrus leaves challenged with *P. parasitica* or *A. alternata*, respectively (Table S2 and Figure S3b,e).

In bacterial stress (subset 3), the stability of expression was evaluated under two experimental conditions: in symptomatic leaves of sweet orange infected with *Ca*. Liberibacter asiaticus versus uninoculated controls, and in leaves 24 h and 7 days post-inoculation with *X. fastidiosa*. *FBOX* and *GAPC2* were calculated to be the most stable genes in geNorm and *ACT2* was considered the most stable in NormFinder (Figure 2d and Table 2). Considering only samples related to *Ca*. L. asiaticus treatment, the *DIM1*/*GAPC2* gene pair was considered the most stable in geNorm, followed by *FBOX*, while *TUB* was ranked as the worst. In general, all candidate genes except *CyP* and *TUB* presented relatively low *M* values (*M*≤0.5) and could be selected as reference genes for studies of gene expression in citrus infected with *Ca*. L. asiaticus (Table S2 and Figure S3a).

For the treatment with *X. fastidiosa*, *FBOX* and *SAND* were the best reference genes according to geNorm. Again, *TUB* showed the greatest variation among all the reference genes tested but none had a value of *M* greater than 0.5. In this case, all genes may therefore be candidates for normalization of gene expression levels in citrus challenged with this pathogen (Table S2 and Figure S3c).

Finally, in the fourth subset evaluated (viral stress), *UBC21*/*UPL7* was selected as the most stable pair by GeNorm, while *DIM1*/*FBOX* was the best combination of two genes in NormFinder. Although *TUB* and *CyP* were considered the most variable genes by both programs, none of the candidate genes differed significantly in stability of expression (Figure 2e and Tables 2, 3). In all treatments individually evaluated, the inclusion of a third gene for more accurate normalization was not required (Figure S4).

In summary, a comparison of geNorm and NormFinder suggested that *FBOX*, *GAPC2*, *SAND* and *UPL7* were the most stable reference genes for all samples and subsets tested in this study. We suggest that these genes could be used as reference genes for accurate transcript normalization in citrus.

### Validation of the selected reference genes

**Figure 4:**
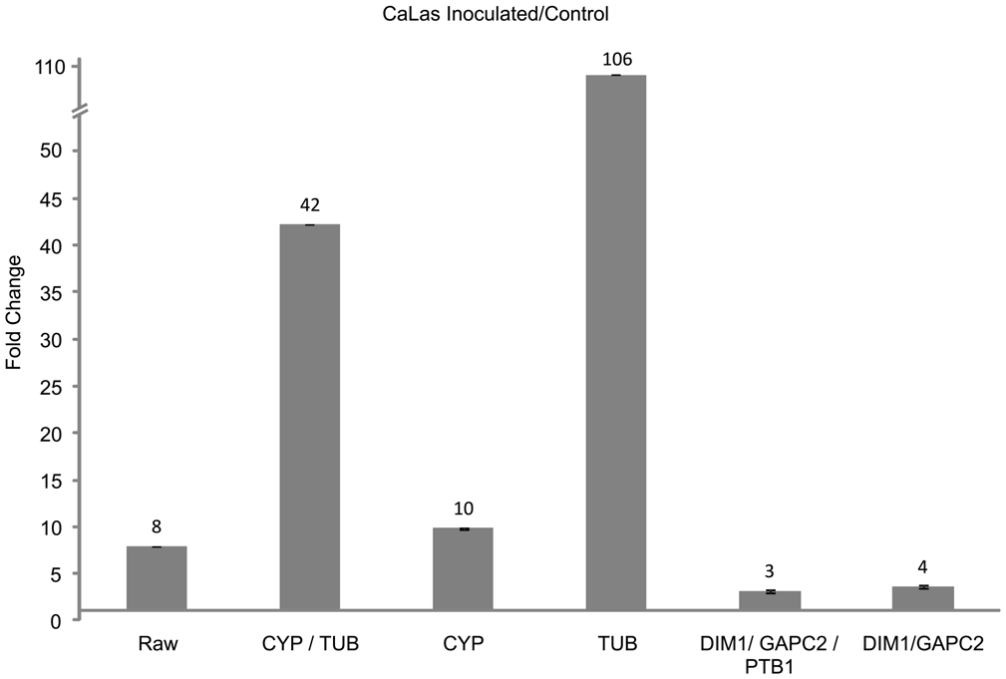
Transcript level of the *WRKY70* transcription factor gene in citrus under infection with *C.* Liberibacter asiaticus. Error bars show mean standard error calculated from two biological replicates. Normalization factors were calculated as the geometric mean of the expression levels of the three most stable reference genes (*DIM1*, *GAPC2* and *PTB1*) and the two most unstable (*CYP* and *TUB*). A control uninoculated sample was used as calibrator.

In order to validate the selected reference genes, the relative expression level of the gene encoding transcription factor WRKY70 was evaluated in plants infected with *Ca*. L. asiaticus. In *Arabidopsis*, this gene acts as an activator of salicylic acid-dependent defense genes and a repressor of jasmonic acid-regulated genes. We also found that citrus *WRKY70* is an important gene in response to infection with *Ca*. L. asiaticus and americanus. According to our microarray analysis, *WRKY70* was upregulated in symptomatic sweet orange plants in relation to uninoculated control plants (Mafra et al., unpublished data). Primer design, RT-qPCR and amplification efficiency calculus were performed as described above, and primer sequences are listed in Table S1. *WRKY70* was normalized to the three most stable candidate reference genes (*DIM1*, *GAPC2* and *PTB11*) and the two least stable (*CYP* and *TUB*) as determined by geNorm analysis. RT-qPCR analysis showed that the expression level of *WRKY70* transcript significantly increased during symptoms in relation to uninoculated controls (fold change, FC = 3.19) (Figure 4). Increased expression of this transcript corroborates our microarray expression data in plants infected with *Ca*. L. americanus (FC = 5.13). A similar expression pattern was described by Albrecht & Bowman (2008), who found that *WRKY70* transcript accumulated in sweet orange leaves infected with *Ca*. L. asiaticus both at 5–9 and 13–17 weeks after inoculation (FC = 1.9 and 2.3, respectively) [19]. In order to demonstrate the effect of using different normalization genes to estimate relative accumulation, we used the three most stable genes (NF_3_) and the two least stable (NF_2_) to calculate normalization factors. As shown in Figure 4, the use of *TUB* only as a reference gene or grouped with CyP to normalize the transcript level of *WRKY70* led to an increase in the fold change (FC = 42 and FC = 106, respectively) compared to the values obtained with the two or three most stable reference genes. These results were expected because of the high variability of M calculated by geNorm for the *Ca*. L. asiaticus condition, when *TUB* was included among the 15 genes assessed (*M* = 0.876) (Table S2).

## Discussion

RT-qPCR has become an important tool to understand gene expression in several biological systems. For accurate RT-qPCR measurements, endogenous reference genes are used as internal controls. An appropriate reference gene should be expressed with minimal change regardless of the experimental conditions. Because there is no reference gene that is universally stable in expression, it is necessary to identify candidate genes specifically chosen for transcript normalization for the conditions under study [2], [44].

Here, we evaluated the stability of expression of eleven novel and four traditional reference genes in citrus from different tissues and under different biotic stresses. Our analysis in geNorm and NormFinder showed some differences, especially in the top ranked genes, but both programs very consistently excluded the same genes as showing unstable expression patterns. This apparent divergence probably reflects differences in the statistical algorithms. The NormFinder program employs a model-based variance estimation approach to identify genes suitable for normalization. In practice, it estimates both the intra- and inter-group variation and combines them into a stability value. This model-based approach ranks the top genes with minimal estimated inter- and intra-group variation. In contrast, the pairwise approach performed by geNorm selects two genes with the highest degree of similarity in expression profile and the lowest intra-group variation. For this reason, it is not surprising that the two algorithms differ in the ranking of the best candidate genes. This divergence in results obtained by the two methods was highlighted in the original paper describing the NormFinder strategy [26]. Discrepancies between NormFinder and geNorm were also demonstrated by other studies [4], [5], [32], [52]. This approach could be problematic if co-regulated genes exhibit similar expression profiles and thus, might be preferentially top ranked [24]. Under viral stress, we found that the *UBC21*/*UPL7* pair was identified as the most stable by geNorm, followed by *UBC9*. The *UBC21* and *UBC9* genes encode ubiquitin-conjugating enzymes belonging to the E2 class, whereas *UPL7* encodes a ubiquitin-protein ligase grouped into the E3 class. Ubiquitin conjugation is a protein modification that occurs in a multistep reaction, sequentially involving an E1 enzyme (ubiquitin-activating enzyme), an E2 enzyme and an E3 enzyme [45]. In *A. thaliana*, it was estimated that there are two E1 proteins, 37 E2 proteins and more than 1,300 predicted E3 proteins [46]. Although E2 and E3 proteins participate in the same pathway, there is no evidence that *UPL7* and *UBC21* interact directly and may be co-regulated. Indeed, only in viral stress and when comparing different organs were these two genes ranked among the top three, while in other subsets they presented intermediate stability values. Moreover, in *Arabidopsis*, these genes were not top ranked by geNorm software, but occupied close positions in the ranking [21].

Our results demonstrated that *FBOX*, *GAPC2*, *SAND* and *UPL7* were the most stably expressed reference genes in all samples and subsets studied. Nevertheless, the best combination of genes varied significantly depending on experimental condition. This observation reinforces the necessity to assay the stability of expression of candidate genes to select suitable reference genes for reliable normalization in a specific biological assay. Among the top reference genes, *FBOX* was identified as the most stable, followed by *GAPC2* and *SAND*. Our results corroborate a recently published paper by Lilly et al. [47], which tested reference genes for normalization of transcripts from virus-infected *A. thaliana*. They found that *FBOX* and *SAND* showed the most stable transcript accumulation. Similar results in *Arabidopsis* were observed by Remans et al. [23], in which the same two genes, along with *YLS8*, were identified as the best candidates for data normalization (*M*<0.3) in roots and shoots in treatments with cadmium and copper. *FBOX* and *SAND* were also ranked among the top 22 most stable reference genes tested in 79 samples including different developmental stages, organs, tissues and genotypes [21]. In soybean, a possible ortholog of *FBOX* was identified as the most uniformly expressed gene [31]. Additionally, *FBOX* was considered a good reference gene for normalization of floral organs in cotton; however, when all organs were compared, this gene was ranked among the three least stable [34]. Despite slight differences found in different studies, we concluded that *FBOX* is a good candidate gene for normalizing a wide range of tissue and organ samples and different conditions in plants, even though the molecular function and biological process this gene is associated with remain unclear.

*SAND* was ranked in our study as the third most stable reference gene. Similarly, *SAND* was revealed as one of the superior reference genes found for proper normalization in tomato development studies and a set of organs and tissues of buckwheat [30], [48]. Also, *SAND* and *RAN1* were calculated as the most stable pair when the entire dataset was evaluated in petunia, while *GAPC* was the most variable gene (*M* = 1.15). *SAND* was first described in the *Saccharomyces cerevisiae* genome. Later, with the availability of several eukaryotic genomes, homologous sequences were identified in *Caenorhabditis elegans*, *Drosophila melanogaster* and *A. thaliana*. In plants, only one SAND sequence was found in monocots and dicots [49]. Functional studies suggest that the SAND family proteins are involved in late steps of endocytic transport [49], [50]. As suggested by Lilly et al. [47], *SAND* may not be a suitable reference gene in studies of gene expression in response to pathogens that could interfere with vesicle traffic, like viruses.

Genes commonly referred to as housekeeping genes, such as tubulins, actins, *GAPDH*, ribosomal subunits and elongation factors, have been used in several studies in citrus to normalize gene expression data. However, there is a consensus that the use of such genes arbitrarily may result in the misinterpretation of results [6], [51]. In our study, *GAPC2* was selected as the second most stable gene overall. In the entire dataset, *GAPC2* was ranked as the third most stable (*M* = 0.5), and in *Ca*. L. asiaticus infection, *GAPC2* and *DIM1* were considered the best combination by geNorm (*M*<0.1). These results are consistent with citrus leaves subjected to drought stress, for which the *EF1*/*ADP* pair was considered by geNorm and NormFinder to be the best combination of genes, followed by *GAPC2* [38]. Similar results were observed in *Brachypodium*, for which NormFinder considered *GAPC2* among the three most stable genes when comparing different tissues (M = 0.28), treatment with growth hormone (*M* = 0.18), and exposure to heat or cold and high salt or drought stress (*M* = 0.07) [4]. However, our results are in contrast with those of Boava et al. [35], who ranked *GAPDH* among the three genes least stable in all conditions assessed. In petunia, *GAPDH* was again considered the gene least stably expressed when assessed during leaf and flower development [37]. Unlike *GAPC2*, which was shown to be a good reference gene for citrus normalization in different biological contexts, *TUB* was ranked in the last position for different subsets analyzed in our study. Our results corroborate those obtained by Carvalho et al. [38], which considered *TUB* inadequate for transcript normalization in citrus under drought stress.

Actin, another reference gene frequently used in citrus, displayed an intermediate stability pattern in our analysis. Expression instability was also described by Czechowski et al. [21], who found *ACT2* to be the least stably expressed gene among the 27 tested. Stability of *ACT* and *TUB* was also assessed in flax and both were considered unreliable for transcript normalization during flax development [52]. Under drought stress in citrus, *ACT2* also showed unstable transcript abundance. Given these observations, we suggest that both *TUB* and *ACT2* should be carefully evaluated before using them as reference genes for citrus gene expression studies.

Finally, to validate the suitability of the reference genes we identified in this study, we assessed the expression profile of a *WRKY70* homolog in leaves of sweet orange plants infected with *Ca*. L. asiaticus. We demonstrated that the use of the two most variable reference genes (*CYP*/*TUB*) or *TUB* resulted in an increase of the relative transcript abundance of *WRKY70* compared to the normalized expression data obtained using the two or three most stable ones (*DIM1*/*GAPC2*/*PTB1* or *DIM1*/*GAPC2*). These results indicate that the incorrect use of reference genes without validation may introduce bias in the analysis and lead to misinterpretation of data. Matta and collaborators [24] reported similar results in qPCR studies of *Drosophila*, emphasizing the need for validation of the best set of reference genes for each experimental condition tested.

In summary, we evaluated several suitable reference genes in different citrus organs and following different biotic stresses. We also identified novel reference genes that outperformed housekeeping genes commonly used in citrus and showed that some of these housekeeping genes could be inadequate for transcript normalization under particular experimental conditions. We propose *FBOX*, *SAND*, *GAPC2* and *UPL7* as good candidate genes to be tested as reference genes for normalization in citrus gene expression studies. In addition, we provide a list of twelve genes with the potential to be good reference genes. This work constitutes the first systematic study in citrus to identify and validate optimal reference genes for RT-qPCR normalization with consideration of different tissues, genotypes and biotic stress conditions.

## Materials and Methods

### Plant materials and experimental conditions

#### Biotic stress assays

The following citrus species and hybrids were included in the evaluation: sweet orange (*C. sinensis* L. Osbeck), Ponkan mandarin (*C. reticulata* Blanco), clementine (*C. clementina* hort. ex Tanaka), Sunki mandarin (*C. sunki* (Hayata) hort. ex Tanaka), Cleopatra mandarin (*C. reshni* hort. ex Tanaka), Murcot tangor (*C. sinensis* L. Osb.×*C. reticulata* Blanco), and *P. trifoliata*. (L.) Raf. All experiments testing a biotic stress were conducted in a greenhouse or growth chamber and are summarized in Table 4. The samples infected with systemic (*X. fastidiosa*, *Ca*. L. asiaticus, and *A. alternata*), or nonsystemic pathogens (CiLV-C, *P. parasitica*) were collected and immediately frozen in liquid nitrogen. For a detailed description of each biotic stress assay, see File S1 in supporting information.

**Table 4:** Summary of biotic stress assays used to select candidate citrus genes for normalization in RT-qPCR.

Biotic stress	Pathogen	Citrus species/Age	Challenge	Sampling (ai^(1)^)	Tissue
*Huanglongbing*	*Candidatus* Liberibacter asiaticus	Sweet orange/Six months after grafting	Grafting with infected budwood	Symptoms ∼150 d	Leaf
CVC^(2)^	*Xylella fastidiosa*	Sweet orange and Ponkan mandarin/Six months after grafting	Needle inoculation of bacterial suspension (10^10^ cells mL^−1^)	24 h and 7 d	Leaf
Leprosis	*Citrus leprosis virus* (CiLV-C)	Sweet orange and Murcot tangor/Six months after grafting	Infested with viruliferous or non-viruliferous mite vector	48 h	Leaf
Brown spot	*Alternaria alternata*	Sweet orange, Murcot tangor, Clementine and Cleopatra mandarin/Three months after grafting	Conidial suspension (10^6^ spores/mL)	6 and 12 h	Leaf
Gummosis	*Phytophthora parasitica*	Sunki mandarin *Poncirus trifoliata*/Ten months after grafting	Mycelial disk	48 h	Leaf

ai^(1)^ = after inoculation.

CVC^(2)^ = Citrus variegated chlorosis.

#### Plant tissues, organs and developmental stages used for sampling

Three 15-year-old ‘Valencia’ orange (*C. sinensis* L. Osbeck) plants grafted onto Cleopatra mandarin (*C. reticulata* Blanco) were used. These trees are cultivated in an experimental field of the Centro de Citricultura Sylvio Moreira, located in Cordeirópolis, São Paulo state, Brazil. Samples of adult leaves, branches, fruitlets (8 mm length) open flowers, and flower buds (5 mm and 10 mm length) were collected during bloom. We also collected meristem samples during winter and early spring. Samples were transferred to liquid nitrogen and stored at −80°C until required.

### Total RNA isolation and cDNA synthesis

About 200 mg of tissue was ground to a fine powder in liquid nitrogen using a mortar and pestle. Total RNA was extracted using an RNeasy Plant Mini Kit (Qiagen) according to the manufacturer's instructions with minor modifications. Genomic DNA contamination was removed by digestion in the RNeasy columns with recombinant DNAse I (Qiagen). Total RNA concentration and purity were determined from the ratio of absorbance readings at 260 and 280 nm using a Nanodrop ND8000 spectrophotometer (Nanodrop Technologies), and RNA integrity was tested in a denaturing agarose gel. Reverse transcription was performed with 1 µg of total RNA in a total volume of 20 µL with oligo(dT) primer using Revertaid H-Minus reverse transcriptase (Fermentas). The final cDNA products were diluted 50-fold prior to use in RT-qPCR.

### Selection of potential reference genes in citrus and primer design

The 15 candidate genes evaluated in this experiment were selected from the CitEST (http://limonia.centrodecitricultura.br/blast/blast.html) and HarvEST (http://www.harvest-web.org/) citrus databases according to meeting one or more of the following criteria: (1) reference genes traditionally used in citrus for transcript normalization; (2) reference genes described in the literature for RT-qPCR normalization in Swingle citrumelo (*C. paradise×P. trifoliata*) under drought stress [38]; and (3) citrus homologues of reference genes tested for transcript level normalization and quantification in *Arabidopsis* [21]. BLASTN with a default setting was used to search for citrus coding sequences with high similarity (*E-value*≤1e-90) to *Arabidopsis* genes. Primers were designed with Primer 3 (http://frodo.wi.mit.edu/primer3/) and Oligo Explorer 1.1.2 software tools (http://www.uku.fi/~kuulasma/OligoSoftware/) with the following parameters: Tm around 60°C and amplicon length of 90 to 120 bp, yielding primer sequences with a length of 19 to 23 nucleotides with an optimum at 20 nucleotides, and a GC content of 45 to 60%. Primers were also designed as much as possible to allow the amplification of transcript isoforms from all citrus genotypes. The specificity of the resulting primer pair sequences was checked against the *Arabidopsis* transcript database using TAIR WU-BLAST2 (www.arabidopsis.org/wublast/index2.jsp). Amplicon specificity was checked by 2% (w/v) agarose gel electrophoresis and by melting-curve analysis. The sequence of the 15 amplicons was confirmed by sequencing (data not shown). PCR products were cloned into pGEM-T Easy vector and sequenced using an Applied Biosystems Model 3730 capillary DNA sequencer.

### RT-qPCR conditions and statistical analysis

RT-qPCR was performed in a 96-well optical plate with an ABI PRISM 7500 FAST sequence detection system (Applied Biosystems). The reaction mixture contained 9 µL 2x FAST SYBR Green Master Mix reagent (Applied Biosystems), 3 µL diluted cDNA (1∶50), 120 or 150 nM of each gene-specific primer pair in a final volume of 25 µL. The following standard thermal profile was used for all amplifications: 95°C for 20 sec followed by 40 cycles of 95°C for 3 sec, and 60°C for 30 sec. All assays were performed using three technical replicates and a non-template control, as well as two or three biological replicates. To analyze dissociation curve profiles, the following program was run after the 40 cycles of PCR: 95°C for 15 sec followed by a constant increase in temperature between 60 and 95°C. Primer efficiency for each experimental set was estimated using an algorithm in Real-time PCR Miner software (http://www.miner.ewindup.info/) that calculates primer efficiency and quantification cycle (Cq) values based on the kinetics of individual reactions without the need for a standard curve. Cq values, determined by the second derivative maximum for each biological sample, were converted into non-normalized relative quantities using the formula *Q = E^ΔCq^*, where *E* represents the arithmetic mean of efficiency of all samples for each gene, and *ΔCq* represents the difference between the arithmetic mean Cq value across all samples for this gene, and the Cq value of the sample in question, as recommended by Hellemans et al. [43]. These quantities were imported into geNorm v3.5 (medgen.ugent.be/∼jvdesomp/geNorm/) [25] and NormFinder (www.mdl.dk/publicationsnormfinder.htm) [26] for reference gene selection. First, we performed a global analysis composed of all biological samples in geNorm. Considering the heterogeneity of treatments, we then analyzed each experimental condition individually in an attempt to identify specific reference genes according to the treatments. Finally, once NormFinder calculated both inter- and intra-group variation in the expression stability, thus identifying the best combination of reference genes, we established four subsets composed of the following treatments: tissue or organ (n = 24); viral stress (n = 18), fungal or oomycete stress (n = 48), and bacterial stress (n = 32). These subsets were then analyzed by both geNorm and NormFinder.

## Supporting Information

Figure S1 **RT-qPCR amplification specificity of the15 reference genes.** Amplification fragments were separated by 2% agarose gel electrophoresis. UNK: unknown protein.
(TIF)

Figure S2 **Dissociation curve data for the 15 reference genes tested.**
(TIF)

Table S1 **Primer sequences, optimized concentration, amplicon length and mean efficiencies calculated by Miner.**
(XLS)

Table S2 **Expression stability for each individual treatment determined by geNorm.** M stability values were calculated by geNorm for six treatments in order to find the most stable specific reference genes under each of the conditions tested.
(XLS)

Figure S3 **Reference genes ranked according to their expression stability as determined by geNorm for each experimental condition.** A lower M value indicates more stable expression. The ranking of the reference genes is in Table S1. (a) *C.* Liberibacter asiaticus infection, (b) *A. alternata* infection, (c) *X. fastidiosa* infection, (d) CiLV-C infection, (e) *P. parasitica* infection.
(TIF)

Figure S4 **Pairwise variation (V) to determine the optimal number of reference genes for each experimental condition.** The ranking of the reference genes is in Table S1. (a) *C.* Liberibacter asiaticus infection, (b) *A. alternata* infection, (c) *X. fastidiosa* infection, (d) CiLV-C infection, (e) *P. parasitica* infection.
(TIF)

File S1 **Detailed description of each biotic stress assay used in this study.**
(DOC)

## References

[1] Gachon C, Mingam A, Charrier B (2004). Real-time PCR: what relevance to plant studies?. J Exp Bot.

[2] Wong ML, Medrano JF (2005). Real-time PCR for mRNA quantitation.. Biotechniques.

[3] Nolan T, Hands RE, Bustin SA (2006). Quantification of mRNA using real-time PCR.. Nat Protoc.

[4] Hong SY, Seo PJ, Yang MS, Xiang F, Park CM (2008). Exploring valid reference genes for gene expression studies in *Brachypodium distachyon* by real-time PCR.. BMC Plant Biol.

[5] Wan H, Zhao Z, Qian C, Sui Y, Malik AA (2010). Selection of appropriate reference genes for gene expression studies by quantitative real-time polymerase chain reaction in cucumber.. Anal Biochem.

[6] Gutierrez L, Mauriat M, Guénin S, Pelloux J, Lefebvre JF (2008). The lack of a systematic validation of reference genes: A serious pitfall undervalued in reverse transcription-polymerase chain reaction (RT-PCR) analysis in plants.. Plant Biotechnol J.

[7] Liu Q, Xu J, Liu Y, Zhao X, Deng X (2007). A novel bud mutation that confers abnormal patterns of lycopene accumulation in sweet orange fruit (*Citrus sinensis* L. Osbeck).. J Exp Bot.

[8] Huerta L, Forment J, Gadea J, Fagoaga C, Peña L (2008). Gene expression analysis in citrus reveals the role of gibberellins on photosynthesis and stress.. Plant Cell Environ.

[9] Liu Q, Zhu A, Chai L, Zhou W, Yu K (2009). Transcriptome analysis of a spontaneous mutant in sweet orange [*Citrus sinensis* (L.) Osbeck] during fruit development.. J Exp Bot.

[10] Chai L, Ge X, Xu Q, Deng X (2011). CgSL2, an S-like RNase gene in ‘Zigui shatian’ pummelo (*Citrus grandis* Osbeck), is involved in ovary senescence.. Mol Biol Rep.

[11] Miao HX, Qin YH, Silva JT, Ye ZX, Hu GB (2011). Cloning and expression analysis of S-RNase homologous gene in *Citrus reticulata* Blanco cv. Wuzishatangju.. Plant Sci.

[12] Zheng TG, Qiu WM, Fan GE, Zheng BB, Guo WW (2011). Construction and characterization of a cDNA library from floral organs and fruitlets of *Citrus reticulata*.. Biol Plant.

[13] Endo T, Shimada T, Fujii H, Omura M (2006). Cloning and characterization of 5 MADS-box cDNAs isolated from citrus fruit tissue.. Sci Hort.

[14] Cernadas RA, Camillo LR, Benedetti CE (2008). Transcriptional analysis of the sweet orange interaction with the citrus canker pathogens *Xanthomonas axonopodis* pv. *citri* and *Xanthomonas axonopodis* pv. *aurantifolii*.. Mol Plant Pathol.

[15] Nishikawa F, Endo T, Shimada T, Fujii H, Shimizu T (2009). Differences in seasonal expression of flowering genes between deciduous trifoliate orange and evergreen Satsuma mandarin.. Tree Physiol.

[16] Sharifi-Sirchi GR, Beheshti B, Hosseinipour A, Mansouri M (2011). Priming against Asiatic citrus canker and monitoring of PR genes expression during resistance induction.. Afr J Biotechnol.

[17] Tan FC, Swain SM (2007). Functional characterization of *AP3*, *SOC1* and *WUS* homologues from citrus (*Citrus sinensis*).. Physiol Plant.

[18] Fan J, Chen C, Brlansky RH, Gmitter FG, Li Z-G (2010). Changes in carbohydrate metabolism in *Citrus sinensis* infected with ‘*Candidatus* Liberibacter asiaticus’.. Plant Pathol.

[19] Albrecht U, Bowman KD (2008). Gene expression in *Citrus sinensis* (L.) Osbeck following infection with the bacterial pathogen *Candidatus* Liberibacter asiaticus causing Huanglongbing in Florida.. Plant Sci.

[20] Volkov RA, Panchuk II, Schoffl F (2003). Heat-stress-dependency and developmental modulation of gene expression: the potential of house-keeping genes as internal standards in mRNA expression profiling using real-time RT-PCR.. J Exp Bot.

[21] Czechowski T, Stitt M, Altmann T, Udvardi MK, Scheible WR (2005). Genome wide identification and testing of superior reference genes for transcript normalization in Arabidopsis.. Plant Physiol.

[22] Nicot N, Hausman JF, Hoffmann L, Evers D (2005). Housekeeping gene selection for real-time RT-PCR normalization in potato during biotic and abiotic stress.. J Exp Bot.

[23] Remans T, Smeets K, Opdenakker K, Mathijsen D, Vangronsveld J (2008). Normalisation of real-time RT-PCR gene expression measurements in *Arabidopsis thaliana* exposed to increased metal concentrations.. Planta.

[24] Matta BP, Bitner-Mathé BC, Alves-Ferreira M (2011). Getting real with real-time qPCR: a case study of reference gene selection for morphological variation in *Drosophila melanogaster* wings.. Dev Genes Evol.

[25] Vandesompele J, De Preter K, Pattyn F, Poppe B, Van Roy N (2002). Accurate normalization of real-time quantitative RT-PCR data by geometric averaging of multiple internal control genes.. Genome Biol.

[26] Andersen CL, Jensen JL, Orntoft TF (2004). Normalization of real-time quantitative reverse transcription-PCR data: a model-based variance estimation approach to identify genes suited for normalization, applied to bladder and colon cancer data sets.. Cancer Res.

[27] Reid KE, Olsson N, Schlosser J, Peng F, Lund ST (2006). An optimized grapevine RNA isolation procedure and statistical determination of reference genes for real-time RT-PCR during berry development.. BMC Plant Biol.

[28] Jain M, Nijhawan A, Tyagi AK, Khurana JP (2006). Validation of housekeeping genes as internal control for studying gene expression in rice by quantitative real-time PCR.. Biochem Biophys Res Commun.

[29] Li QF, Sun SSM, Yuan DY, Yu HX, Gu MH (2010). Validation of candidate reference genes for the accurate normalization of real-time quantitative RT-PCR data in rice during seed development.. Plant Mol Biol Rep.

[30] Expósito-Rodríguez M, Borges AA, Borges-Pérez A, Pérez JÁ (2008). Selection of internal control genes for quantitative real-time RT-PCR studies during tomato development process.. BMC Plant Biology.

[31] Libault M, Thibivilliers S, Bilgin DD, Radwan O, Benitez M (2008). Identification of four soybean reference genes for gene expression normalization.. Plant Genome.

[32] Cruz F, Kalaoun S, Nobile P, Colombo C, Almeida J (2009). Evaluation of coffee reference genes for relative expression studies by quantitative real-time RT-PCR.. Mol Breeding.

[33] Silveira ED, Alves-Ferreira M, Guimarães LA, Silva FR, Carneiro VTC (2009). Selection of reference genes for quantitative real-time PCR expression studies in the apomictic and sexual grass *Brachiaria brizantha*.. BMC Plant Biol.

[34] Artico S, Nardeli SM, Brilhante O, Grossi-de-Sá M, Alves-Ferreira M (2010). Identification and evaluation of new reference genes in *Gossypium hirsutum* for accurate normalization of real-time quantitative RT-PCR data.. BMC Plant Biol.

[35] Boava LP, Laia ML, Jacob TR, Dabbas KM, Gonçalves JF (2010). Selection of endogenous genes for gene expression studies in Eucalyptus under biotic (*Puccinia psidii*) and abiotic (acibenzolar-S-methyl) stresses using RT-qPCR.. BMC Res Notes.

[36] Wan H, Zhao Z, Qian C, Sui Y, Malik AA (2010). Selection of appropriate reference genes for gene expression studies by quantitative real-time polymerase chain reaction in cucumber.. Anal Biochem.

[37] Mallona I, Lischewski S, Weiss J, Hause B, Egea-Cortines M (2010). Validation of reference genes for quantitative real-time PCR during leaf and flower development in *Petunia hybrida*.. BMC Plant Biol.

[38] Carvalho K, de Campos MK, Pereira LF, Vieira LG (2010). Reference gene selection for real-time quantitative polymerase chain reaction normalization in “Swingle” citrumelo under drought stress.. Anal Biochem.

[39] Yan J, Yuan F, Long G, Qin L, Deng Z (2011). Selection of reference genes for quantitative real-time RT-PCR analysis in citrus.. Mol Biol Rep.

[40] Boava LP, Cristofani-Yaly M, Mafra VS, Kubo K, Kishi LT (2011). Global gene expression of *Poncirus trifoliata*, *Citrus sunki* and their hybrids under infection of *Phytophthora parasitica*.. BMC Genomics.

[41] Talon M, Gmitter FG (2008). Citrus genomics.. Int J Plant Genomics.

[42] Iglesias DJ, Cercós M, Colmenero-Flores JM, Naranjo MA, Ríos G (2007). Physiology of citrus fruiting.. Braz J Plant Physiol.

[43] Hellemans J, Mortier G, De Paepe A, Speleman F, Vandesompele J (2007). qBase relative quantification framework and software for management and automated analysis for real-time quantitative PCR data.. Genome Biol.

[44] Hruz T, Wyss M, Docquier M, Pfaffl MW, Masanetz S (2011). RefGenes: identification of reliable and condition specific reference genes for RT-qPCR data normalization.. BMC Genomics.

[45] Kraft E, Stone SL, Ma L, Su N, Gao Y (2005). Genome analysis and functional characterization of the E2 and RING-type E3 ligase ubiquitination enzymes of Arabidopsis.. Plant Physiol.

[46] Vierstra RD (2003). The ubiquitin/26S proteasome pathway, the complex last chapter in the life of many plant proteins.. Trends Plant Sci.

[47] Lilly ST, Drummond RSM, Pearson MN, MacDiarmid RM (2011). Identification and validation of reference genes for normalization of transcripts from virus-infected *Arabidopsis thaliana*.. Mol Plant Microbe Interact.

[48] Demidenko NV, Logacheva MD, Penin AA (2011). Selection and validation of reference genes for quantitative real time PCR in buckwheat (*Fagopyrum esculentum*) based on transcriptome sequence data.. PLoS One.

[49] Cottage A, Mullan L, Portela MBD, Hellen E, Carver T (2004). Molecular characterization of the SAND protein family: a study based on comparative genomics, structural bioinformatics and phylogeny.. Cell Mol Biol Lett.

[50] Poteryaev D, Spanga A (2005). A role of SAND-family proteins in endocytosis.. Biochem Soc Trans.

[51] Guénin S, Mauriat M, Pelloux J, Wuytswinkel OV, Bellini C (2009). Normalization of qRT-PCR data: the necessity of adopting a systematic, experimental conditions-specific, validation of references.. J Exp Bot.

[52] Huis R, Hawkins S, Neutelings G (2010). Selection of reference genes for quantitative gene expression normalization in flax (*Linum usitatissimum* L.).. BMC Plant Biology.

