# Supplementary figures and images for: Reference Genes for Accurate Transcript Normalization in Citrus Genotypes under Different Experimental Conditions
Source: PLoS One. 2012 Feb 9;7(2):e31263. doi: 10.1371/journal.pone.0031263 (PMC3276578; doi:10.1371/journal.pone.0031263)

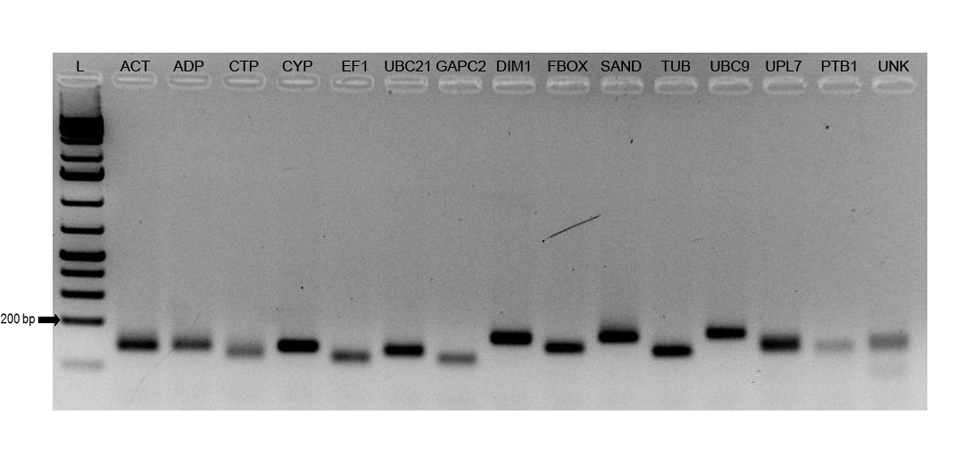

Supplement: Figure S1 — RT-qPCR amplification specificity of the15 reference genes. Amplification fragments were separated by 2% agarose gel electrophoresis. UNK: unknown protein. (TIF) [file pone.0031263.s001.tif]

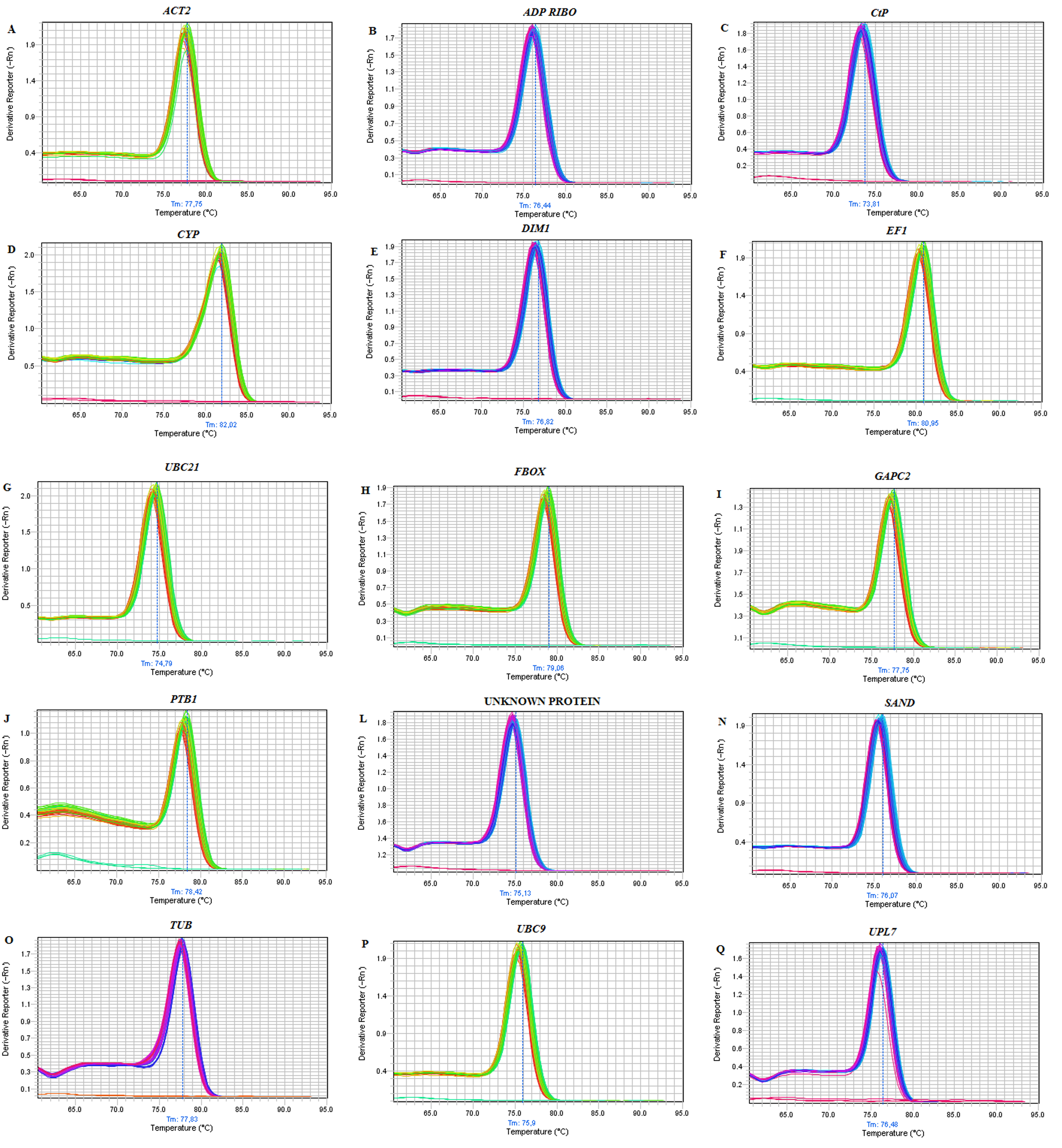

Supplement: Figure S2 — Dissociation curve data for the 15 reference genes tested. (TIF) [file pone.0031263.s002.tif]

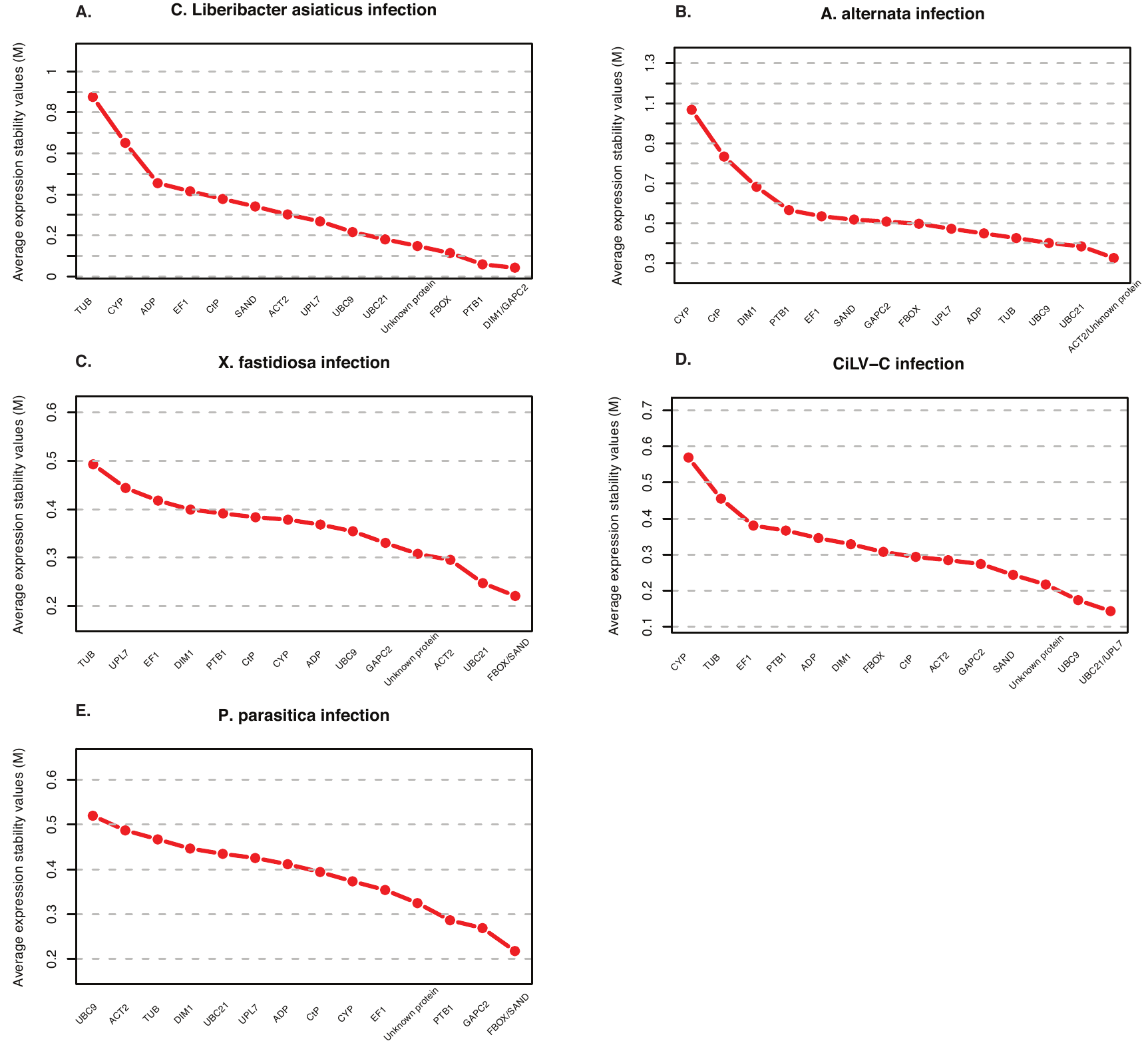

Supplement: Figure S3 — Reference genes ranked according to their expression stability as determined by geNorm for each experimental condition. A lower M value indicates more stable expression. The ranking of the reference genes is in Table S1. (a) C. Liberibacter asiaticus infection, (b) A. alternata infection, (c) X. fastidiosa infection, (d) CiLV-C infection, (e) P. parasitica infection. (TIF) [file pone.0031263.s005.tif]

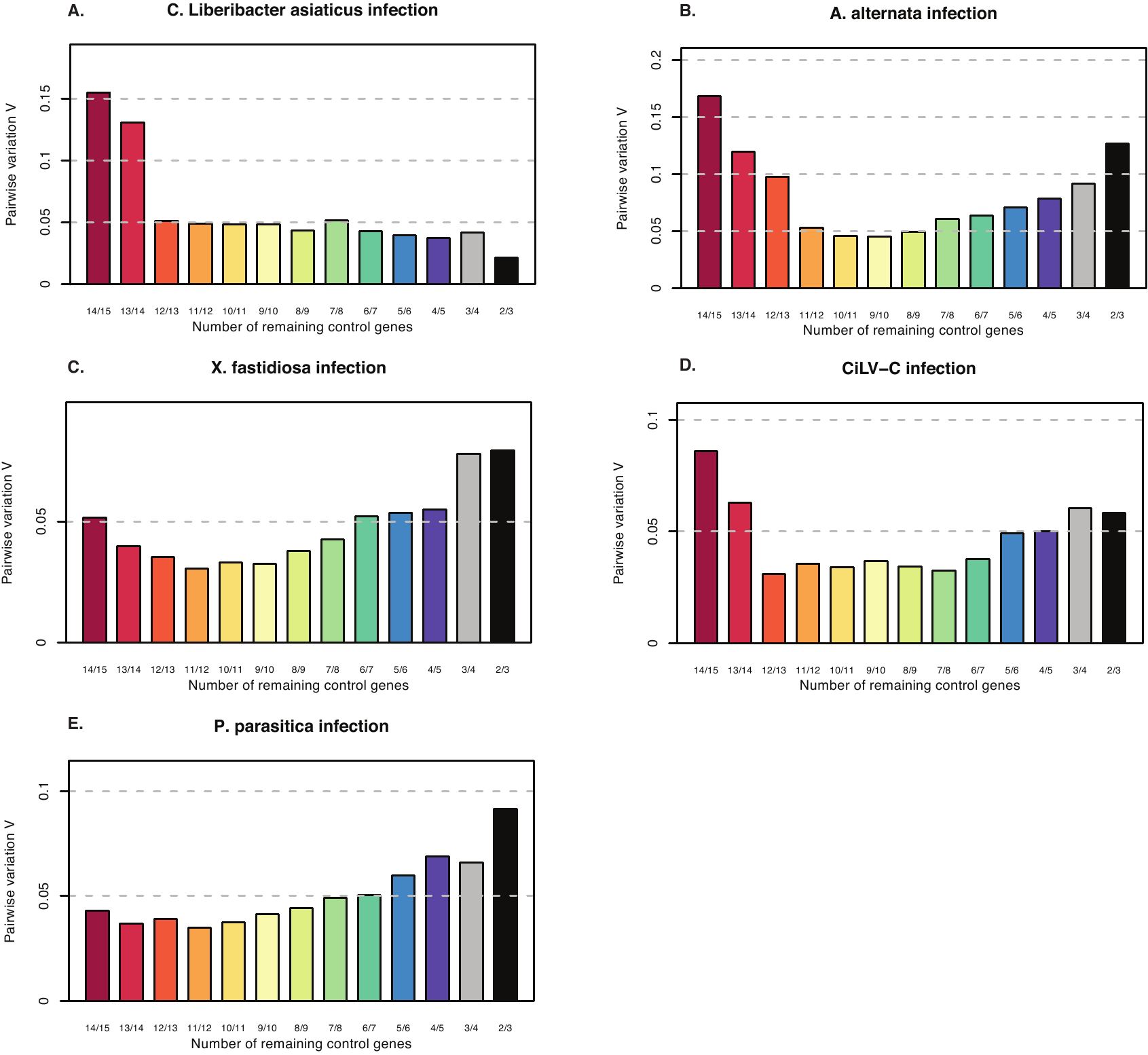

Supplement: Figure S4 — Pairwise variation (V) to determine the optimal number of reference genes for each experimental condition. The ranking of the reference genes is in Table S1. (a) C. Liberibacter asiaticus infection, (b) A. alternata infection, (c) X. fastidiosa infection, (d) CiLV-C infection, (e) P. parasitica infection. (TIF) [file pone.0031263.s006.tif]
