# Supplementary material for: Reference Genes for Accurate Transcript Normalization in Citrus Genotypes under Different Experimental Conditions
Source: PLoS One. 2012 Feb 9;7(2):e31263. doi: 10.1371/journal.pone.0031263 (PMC3276578; doi:10.1371/journal.pone.0031263)
Supplement: File S1 — Detailed description of each biotic stress assay used in this study. (DOC) [file pone.0031263.s007.doc]

**Plant Materials and experimental conditions**

**Biotic stress assays:**

***Candidatus* Liberibacter asiaticus (*Ca*. L. asiaticus) inoculation**

Experiments were performed using three greenhouse-grown six-month-old ‘Pera’ sweet orange plants grafted onto Rangpur lime (*C. limonia*). Five plants were inoculated with infected bark pieces onto the rootstock portion of each plant, and two healthy plants grown at the same conditions were maintained as control plants. Inoculated plants were monthly monitored by end-time PCR until the presence of the pathogen, approximately 150 days post-infection. Plants were pruned to promote new leaf growth and transferred to the growth chamber with a controlled temperature of 22-24° C. Among five plants infected, only two plants developed symptoms. Leaves were collected from two symptomatic (yellowing of leaves) and two healthy plants and stored at -80° C.

**Citrus leprosis virus C (CiLV-C) inoculation**

Experiments were performed in the greenhouse using nine ‘Pêra’ sweet orange (*C. sinensis* L. Osbeck) and nine Murcott tangor (*C. sinensis x C. reticulata*), susceptible and tolerant to CiLV-C, respectively, grafted onto Rangpur lime (*C. limonia*). Three plants were infested with viruliferous, three with non-viruliferous *Brevipalpus phoenicis* mite vector for CiLV-C, and three plants to each genotype were used as negative controls. Leaves were collected 48 hours post infestation and stored at -80° C.

***Xylella* *fastidiosa* inoculation**

Experiments with *X. fastidiosa* were performed in the greenhouse using nine ‘Pera’ sweet orange (*C. sinensis* L. Osbeck) and nine ‘Ponkan’ (*C. reticulata* Blanco), susceptible and resistant to *X. fastidiosa*, respectively, grafted onto Rangpur lime (*C. limonia*). Six plants of each genotype were artificially inoculated by needle prick with 10 µL suspension (10^10^ cells mL^-1^) of *X. fastidiosa* 9a5c strain in PBS buffer. Three negative controls of each genotype were mock inoculated with PBS buffer. Leaves were harvested 24 hours and 7 days post inoculation and stored at -80° C until RNA extraction.

***Phytophthora parasitica inoculation***

Experiments were performed in the greenhouse with six *C. sunki* (Hayata) hort. ex Tanaka and six ‘Rubidoux’ *Poncirus* *trifoliata*, susceptible and resistant to Phytophthora, respectively, grafted onto Rangpur lime (*C. limonia*) rootstock. Three seedlings, each, with six-month-old after grafting were inoculated with *P*. *parasitica* oomycete by a mycelia disc, which was placed onto the center of a cut made in the stem and covered with parafilm. Three plants each were maintained as non-inoculated controls. After 48 hours, leaves were harvested and stored at 80° C.

***Alternaria alternata* inoculation**

Experiments were conducted with three-month old plants of susceptible Murcott tangor (*Citrus sinensis* Osb. x *C. reticulata* Blanco), Pera sweet orange (*C. sinensis* (L.) Osb.), resistant Clementine (*C. clementine* Hort.), and Cleopatra tangerine (*C. reshni* Hort. ex Tanaka), all grafted onto Rangpur lime (*C. limonia* Osb.). The inoculations were performed with the strain 4303 of the tangerine pathotype of *A. alternata*, previously obtained from a severely infected Murcott tangor fruit. Before inoculation, plants were pruned to induce new flush. During all the experiment plants were maintained in a growing chamber at 27 ± 1º C, 75 ± 5% relative humidity (RH) under constant cool fluorescent light. For inoculation, conidial suspension (10^6^ spores/ mL) was produced and sprayed onto young leaves (3-5 cm). Leaves were removed 6 h and 12 h post inoculation (pi) and immediately frozen in liquid nitrogen. The experiment was conducted in three biological replicates for each time. Healthy water-inoculated plants were used as control.
